# Structural insights into synthetic ligands targeting A–A pairs in disease-related CAG RNA repeats

**DOI:** 10.1093/nar/gkz832

**Published:** 2019-09-30

**Authors:** Sanjukta Mukherjee, Leszek Błaszczyk, Wojciech Rypniewski, Christoph Falschlunger, Ronald Micura, Asako Murata, Chikara Dohno, Kazuhiko Nakatani, Agnieszka Kiliszek

**Affiliations:** 1 Department of Regulatory Bioorganic Chemistry, The Institute of Scientific and Industrial Research, Osaka University 8-1 Mihogaoka, Ibaraki 567-0047, Japan; 2 Institute of Bioorganic Chemistry, Polish Academy of Sciences, Noskowskiego 12/14, 61-704 Poznan, Poland; 3 Institute of Organic Chemistry and Center for Molecular Biosciences Innsbruck CMBI, Leopold-Franzens University, Innrain 80-82, Innsbruck 6020, Austria

## Abstract

The trinucleotide repeat expansion disorders (TREDs) constitute of a group of >40 hereditary neurodegenerative human diseases associated with abnormal expansion of repeated sequences, such as CAG repeats. The pathogenic factor is a transcribed RNA or protein whose function in the cell is compromised. The disorders are progressive and incurable. Consequently, many ongoing studies are oriented at developing therapies. We have analyzed crystal structures of RNA containing CAG repeats in complex with synthetic cyclic mismatch-binding ligands (CMBLs). The models show well-defined interactions between the molecules in which the CMBLs mimic nucleobases as they form pseudo-canonical base pairs with adenosine residues and engage in extensive stacking interactions with neighboring nucleotides. The binding of ligands is associated with major structural changes of the CAG repeats, which is consistent with results of biochemical studies. The results constitute an early characterization of the first lead compounds in the search for therapy against TREDs. The crystallographic data indicate how the compounds could be further refined in future biomedical studies.

## INTRODUCTION

Microsatellites or short tandem repeats (STR) are highly abundant sequences scattered throughout the human genome (1). The main type of repeats found within genes is the trinucleotide CNG repeats (N stands for one of the four nucleotides). They are characterized by an instability, which can lead to their expansion (2). If the number of CNG repeats exceeds a certain threshold, they become pathogenic (3–5). More than 40 hereditary neurodegenerative human disorders, classified collectively as TREDs (tri-nucleotide repeat expansion disorders), are associated with the expansion of the trinucleotide repeats (3,5). A quarter of them result from the expansion of CAG repeats.

The pathogenesis of TREDs depends on where the repeated sequences are localized in the gene (4). When expansion occurs in the coding region, the repeats are translated into abnormal proteins containing long tracts of glutamine or alanine residues, resulting in the gain or loss of protein function (6). This outcome is observed in Huntington's disease, dentatorubral-pallidoluysian atrophy, spinal and bulbar muscular atrophy, and many spinocerebellar ataxias. When the expanded repeats are localized in the untranslated regions or introns, they are transcribed and fold into stable RNA hairpins (7). They have the ability to sequester RNA-binding proteins and form toxic aggregates, known as nuclear foci (8,9). The resulting decrease of free protein leads to a deregulation of important cellular processes, as in spinocerebellar ataxias, myotonic dystrophies, and fragile X-associated tremor/ataxia syndrome.

Repeat expansion disorders are progressive and incurable, and they are the subject of many ongoing studies to develop therapies targeting the toxic RNAs and proteins (10–17). Diverse approaches have been proposed including oligonucleotide-based agents (siRNAs, shRNAs and antisense oligonucleotides) and small ligands. The latter have several advantages, such as oral delivery allowing effective biodistribution, ease of chemical modification, and cost-effective manufacturing. On the other hand, such small ligands are difficult to develop due to the limited structural information of the target RNA and its overall negative charge, which impedes selectivity toward specific RNA. The major bottleneck is the lack of structural knowledge of RNA–ligand complexes, which would aid the design of potential RNA-binding molecules or the selection of compounds from chemical libraries for high-throughput screening.

In recent years, we have solved the crystal structures of all four CNG repeats (18–22). This allowed a detailed 3D ‘profiling’ of the RNA repeats. Properties of the CNG repeats are determined by the noncanonical base pairs. Each pair interacts in a distinct way, reflected in the hydrogen bonding pattern, helical parameters and electrostatic potential surface. This helped to explain *in vitro* and *in vivo* observations relevant to the biomedical aspects of TREDs.

**Figure 1.**
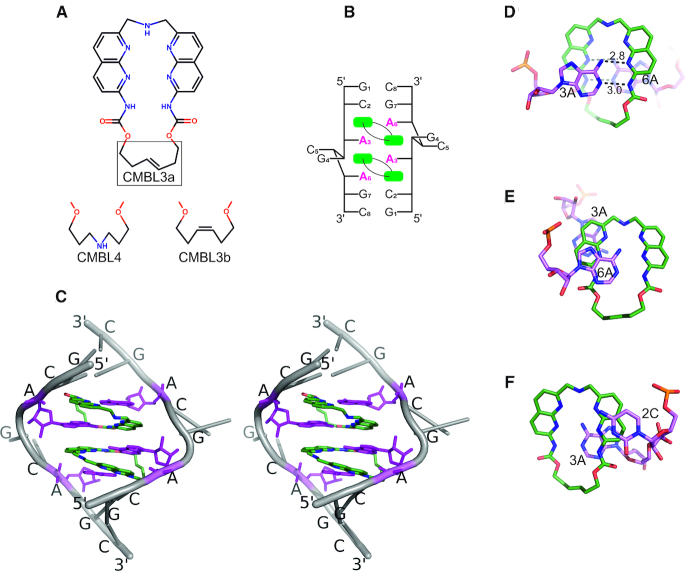
The CMBL ligands and their complexes with RNA. (**A**) Structures of CMBL3a, CMBL3b and CMBL4. (**B**) Secondary structure diagram of the CMBL/RNA complex. (**C**) A stereo view of X-ray structure of CMBL3a with (GCAGCAGC)_2_. Each adenine (*purple*) interacts with one naphthyridine moiety (*green*). The sugar-phosphate backbone and the other residues are indicated in *gray*. (**D**) Hydrogen bonding interactions between CMBL3b and adenosine residues 3A, one from each RNA strand. (**E, F**), Stacking interactions between CMBL3b and residues 3A, 6A of one RNA strand, and 2C and 3A of the other strand.

Subsequently, we have designed a group of small molecules to target noncanonical base-pairs in RNA or mismatches in DNA (23–28). The ligands are composed of two aromatic heterocycles connected by a linker. The aromatic component is designed to form complementary H-bonds with the nucleobase and stack with the neighboring base pair. One type of the aromatic moieties is a 1,8-naphthyridine ring containing two imino groups, which favors guanine as a binding partner (29–31). Recently, we have developed cyclic mismatch-binding ligands (CMBLs) composed of two naphthyridine rings connected by two linkers, which were found to interact with CAG repeats (32,33) (Figure 1A).

Here, we report the structural and biochemical studies of CMBLs in complexes with the CAG repeats. These first crystal structures of RNA repeats with small ligands show major structural changes, as the ligands recognize the noncanonical A-A pairs. The observed induced fit would be difficult to foresee, yet the structures should be insightful for chemists attempting to develop molecules to target RNA.

## MATERIALS AND METHODS

### Crystallization, data collection and refinement

Native GCAGCAGC oligomer was purchased from Future synthesis, while the 2′-methylseleno cytidine phosphoramidite and the oligomer GCAG(2′-SeCH_3_-C)AGC were synthesized according to published protocols (34–36). Complexes of RNA with cyclic bis-naphthyridines were crystallized using the sitting drop vapor diffusion method. Crystallization conditions were determined using sparse-matrix screens. RNA and CMBL ligands were mixed in 1:2 molar ratio. The final concentration of RNA was 0.75 mM. The crystallization drop contained 1.5 μl of the RNA-CMBL complex and 0.75 μl of the appropriate crystallization screen solution. The seleno-derivative RNA was crystallized with CMBL3a using 5 mM MgCl_2_, TRIS buffer at pH 7.5 and 30% PEG 400. Complexes with CMBL3a were obtained from 10 mM MgCl_2_, cacodylate buffer at pH 6.0 and 1 M Li_2_SO_4_. In case of the CMBL3b, the conditions were 20 mM magnesium acetate, cacodylate buffer at pH 6.0 and 1.7 M ammonium sulfate and for CMBL4 2 mM MgCl_2_, MOPS buffer at pH 7.0, Tacsimate and 5 mM hexamine cobalt chloride. Crystals usually appeared within several days.

Most of the X-ray diffraction data were collected at the BESSY synchrotron in Berlin on the BL 14.1 beam line. Only crystals of the CMBL3b complex were measured on the P11 line at PETRA in Hamburg. Single-wavelength anomalous dispersion (SAD) data were collected at the wavelength 0.9756 Å corresponding to the selenium absorption edge. The data were integrated and scaled using the program suite XDS with XDSAPP or xdsgui interface (37,38). Data cut-off was based on CC1/2 statistics or Rmerge and I/Sigma parameters. The structure of CAG(Se)-CMBL3a complex was first solved by the SAD method using the Auto-Rickshaw web server (39). The other structures were solved by molecular replacement using PHASER (40) and the CAG(Se)-CMBL3a structure as the search model. The manual model building was done using Coot (41). Early stages of the refinement were done using the program Refmac5 from the CCP4 program suite, then refinement was carried out with PHENIX (42–44). Restraints for the CMBL ligands and seleno-cytosine was generated by the grade web server developed by Global Phasing (http://grade.globalphasing.org). All pictures were drawn using PyMOL (45). Atomic coordinates of the crystallographic models have been deposited with the Protein Data Bank (accession codes: 6QIQ, 6QIR, 6QIS, 6QIT, 6QIV). The statistics of X-ray data collection and refinement are summarized in Table 1.

**Table 1.** X-ray data collection and refinement

	CAG-CMBL3a(I)	CAG-CMBL3a(II)	CAG-CMBL3b	CAG-CMBL4	CAG(Se)-CMBL3a
**Data collection**	Native	Native	Native	Native	SAD
Space group	*C*2	*P*2_1_2_1_2_1_	*P*2_1_	*P*4_2_2_1_2	*P*6_5_22
Cell parameters (Å, °)	*a* = 77.0, *b* = 46.7, *c* = 54.8, *β*= 129.7	*a* = 45.9, *b* = 51.3, *c* = 118.9	*a* = 35.2 *b* = 51.0, *c* = 39.6 *β* = 104.1	*a* = *b* = 37.9, *c* = 38.7	*a* = *b* = 46.9, *c* = 214.0
Resolution (Å)	1.53	1.99	1.50	2.28	2.52
R_merge_	0.041 (1.29)	0.092 (0.940)	0.056 (0.752)	0.095(0.679)	0.113 (2.873)
*I*/*σ*	11.9 (0.8)	12.2 (2.2)	11.0 (1.5)	13.2 (2.7)	18.3 (1.0)
CC_1/2_	99.9 (87.2)	99.9 (92.3)	99.8 (65)	99.8 (97.6)	99.9 (64.9)
Completeness (%)	97.4 (96.2)	99.9 (99.5)	96.1 (94.3)	99.7 (100.0)	100.0(99.9)
Redundancy	3.5 (3.4)	10.1 (10.4)	2.8 (2.7)	8.2 (9.0)	27.9 (28.5)
**Refinement**					
Overall mean *B*-factor (Å^2^)	39.6	41.1	22.9	44.2	87.6
Number of reflections	22183	25185	20920	1427	8867 (unmerged)
*R*_work_/*R*_free_	17.7/23.3	23.9/27.6	15.0/19.5	19.9/25.4	20.4/22.7
RNA atoms	680	1513	694	170	342
Water molecules	141	126	179	1	6
No. ligands	4	8	4	1	2
RMSD in bonds (Å)	0.010	0.006	0.009	0.008	0.012
RMSD in angles (°)	1.5	1.2	1.7	1.7	2.0
PDB code	6QIR	6QIS	6QIT	6QIV	6QIQ

### UV melting of RNA-ligands

Thermal denaturation profiles of (UCAACAGUUGA)_2_ and (GCAGCAGC)_2_ (5 μM) with or without CMBL3a (20 μM) were carried out in a buffer containing 10 mM sodium cacodylate pH 7.0, 100 mM NaCl and 2% DMSO. Measurements were performed using SHIMADZU UV-2550 UV/Vis spectrometer linked to a Peltier temperature controller. The absorbance of the sample was monitored at 260 nm from 2°C to 85°C with a heating rate of 1°C/min. The melting point for a given experiment was calculated using the median method. RNA oligonucleotides were purchased from Gene Design Inc.

Detailed UV thermal melting studies were performed according to Pasternak and Wengel (2010) on a JASCO V-650 spectrometer equipped with a thermo-programmer (46). The UV melting was performed in a buffer containing 100 mM NaCl, 20 mM sodium cacodylate, and 0.5 mM Na_2_EDTA, pH 7.0. The GCAGCAGC oligomer was prepared in nine different concentrations in the range 10^−5^ to 10^−6^ M. The concentrations of single strand oligomers were calculated from a high-temperature (>80°C) absorbance and single strand extinction coefficients approximated by the nearest-neighbor model. For the thermodynamics study of RNA-ligand complexes, the appropriate CMBL molecule was added to the RNA duplex in a 4:1 molar ratio. The UV absorption versus temperature was measured at 260 nm at the heating rate of 1°C/min in the range 5°C – 95°C. The melting curves were analyzed and the thermodynamic parameters calculated using MeltWin 3.5. The enthalpy (Δ*H*), entropy (Δ*S*) and free energy (Δ*G*_37_), for 37°C, were calculated by two methods: fitting the experimental and theoretical melting curves and linear correlation of the melting temperature 1/*T*_M_ and concentration of RNA (log C_T_). Melting point (*T*_M_) was calculated at the RNA concentration of 10^−4^ M.

### Surface plasmon resonance (SPR) assay

SPR single cycle kinetics assay were performed using Biacore T200 platform. RNA oligomers were immobilized on the Series S sensor chip SA surface using avidin-biotin coupling in 10 mM HEPES and 150 mM NaCl, pH 7.4. 5′-Biotinylated oligonucleotides (Gene Design Inc.) were diluted to 0.2 μM concentration in 10 mM HEPES and 500 mM NaCl and injected with contact time of 840 s and flow rate of 5 μl/min to reach the response of around 500 RU (Response Unit). Blank immobilization was performed in the flow cell 1 to permit reference subtraction. The amount of immobilized RNA was as follows: (CAG)_9_–518 RU, (CCG)_9_–882 RU, (CGG)_9_–482 RU and (CUG)_9_–503 RU. CMBL3a ligand in 1 mM DMSO was diluted in 10 mM HEPES, 150 mM NaCl, 3.0 mM EDTA, pH 7.4 and 0.005% (v/v) Surfactant P20 such that the final ligand solution contained 5% DMSO. Sensorgrams were obtained in the 0.1–0.5 μM ligand concentration range, 60 μl/min flow rate, 30 min of contact time and 120 s of dissociation time. All sensorgrams were corrected by reference subtraction of blank flow cell response and buffer injection response.

### Circular dichroism (CD)

Circular dichroism measurements were carried out using J-725 CD spectropolarimeter (JASCO). CD titration spectra of (CAG)_9_ (2.5 μM) with variable concentration of CMBL3a (2.5, 5.0, 10, 15, 20, 25, 35, 45, 50 and 55 μM) were measured at ambient temperature in 10 mM sodium cacodylate pH 7.0 and 100 mM NaCl. The apparent dissociation constant (*K*_d_) of (CAG)_9_-CMBL3a complex was calculated by fitting the data to the 1:2 binding isotherm. RNA oligonucleotides were purchased from Gene Design Inc.

## RESULTS

Three cyclic bis-naphthyridines (CMBLs) were used in this study: CMBL3a, CMBL3b and CMBL4 (Figure 1A). In each ligand, the naphthyridines are connected by two linkers. The shorter one consists of an amino group. The longer linker contains two *N*-acyl-amino groups and additional constituents: in CMBL3a it is a *E*-alkene, in CMBL3b it is an *Z*-alkene, and in CMBL4 it is an aminoalkyl moiety. Each ligand was cocrystallized with a GCAGCAGC RNA oligomer, giving crystals suitable for X-ray diffraction experiments.

In total, three X-ray structures consisting of (GCAGCAGC)_2_-CMBL3a, one structure of (GCAGCAGC)_2_-CMBL3b and one (GCAGCAGC)_2_-CMBL4 complex have been analyzed (Table 1). Electron density maps of all of the models are well defined and unambiguous. The ligand molecules were easily identified and modeled into the electron density. The superposition of the duplexes shows that all of the models are similar, having a r.m.s.d. of 1.1 Å. A comparison of the multiple models presents an opportunity to identify the characteristic structural features of the RNA-CMBL complexes while taking into account the possible crystal packing effects.

### The overall structure of the RNA-ligand complex

In all of the models, two CMBL molecules are bound to each RNA duplex. The ligands are located inside the RNA helix and mimic nucleobases. One ligand interacts simultaneously with two adenosine residues located on opposite RNA strands (Figure 1B, C). Each naphthyridine forms a pseudocanonical base pair with an adenine. Four such base pairs are formed in a duplex. They are located in the central part of the helix and are flanked on each side by C–G and G–C pairs. The central guanosine 4G and cytosine 5C residues, which in the unliganded structure form C–G and G–C pairs, are flipped out of the helix (Figure 1C). The flipping is associated with a widening and kinking of the helix, which enables the pseudocanonical CMBL–adenine base pairs to stack one above another. All of the RNA–CMBL complexes show internal symmetry, indicating that the ligands bind to the A–A pairs in the same manner. In addition, the cyclic ligands possess chemical symmetry, and each naphthyridine interacts identically with one adenine nucleobase.

### Pseudocanonical CMBL–adenine pair

Each adenosine residue interacts with CMBL by two hydrogen bonds. One is formed between the N1 imino group of A and the NH of the ligand's amide group. The second H-bond is between the *exo*-amino group of A and the imine group of the naphthyridine ring (Figure 1D). These are the only direct hydrogen bonds between the ligands and the nucleic acid. The other interactions between CMBL and RNA involve stacking of aromatic moieties. One naphthyridine ring is wedged between two adenosine residues (3A and 6A). It forms extensive stacking interactions with the former. It also stacks with the second A but to a lesser extent (Figure 1E). The second naphthyridine is placed between adenosine 3A and cytidine 2C of the other RNA strand and stacks extensively against both of them (Figure 1F).

### Conformation of CMBL ligands

**Figure 2.**
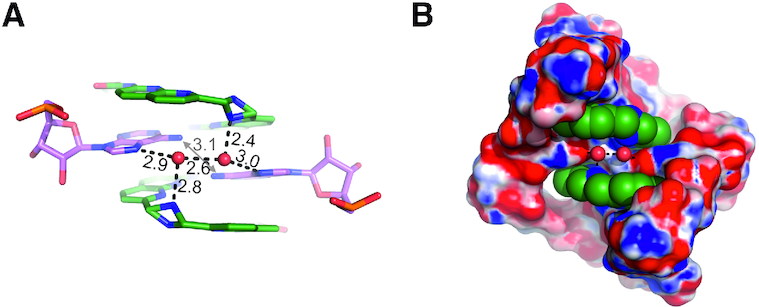
Interactions with solvent molecules. (**A**) The adenosines 3A and CMBL3a form hydrogen bond with two characteristic water molecules (*red spheres*). The shorter linker of the ligand exhibits static disorder with a double conformation. (**B**) The electrostatic potential surface of the CAG repeats. *Blue* is positive, *red* is negative. The CMBL ligand is shown as Van der Waals model.

Each cyclic bis-naphthyridine molecule is in an unfolded conformation (the naphthyridine rings are not stacked) (Figure 1A, C). The imino groups of the aromatic moieties are on the inner side of the ligand and are involved in recognition of the adenosine residues. The short linker consisting of the amino group is in the major groove, while the longer linker is in the minor groove (Figure 1C). In most of the examined structures, the longer spacer exhibits a single well-defined conformation, except for CMBL3b, which shows two conformations around the C=C double bond. The carbonyl oxygen atoms of the spacers point towards the minor groove. They are exposed to the solvent and in some models interact with water molecules. The amino group of the shorter linker points either up or down the helix axis. In most instances, the linker exhibits one of two possible arrangements, but in four cases, it is disordered, showing both conformations (Figure 2A). Despite the variable geometry of the linker, the amino group interacts with a water molecule, which usually at the same time interacts with the Hoogsteen edge of an adenosine residue.

### Structural characteristics of adenosine residues

The flipping out of the 4G and 5C residues and the change of direction of the sugar-phosphate backbone is associated with an unusual C2′-*endo* conformation of the 3A ribose (Supplementary Figure S1). The rearrangement of the RNA chain is stabilized by a H-bond between the 2′OH group of the 3A residue and the phosphate group of 5C. The 6A residue shows the 3′-*endo* sugar puckering, typical of A-RNA.

In the pseudo base pairs, formed between the adenosines and the CMBL ligands, the same functional groups of the adenosines are involved as in the canonical A–U pairs. Additionally, the pairs’ show a propeller twist, as in canonical pairing. This results in a close distance of 3.1 Å between the *exo*-amino groups of the 3A residues from each RNA strand (Figure 2A). Moreover, the 3A residues form a niche in the major groove, exhibiting a negative electrostatic potential due to their N7 imino groups. Two water molecules are located in the niche (Figure 2A, B). Their placing indicates the local hydrogen bonding potential. Each water interacts with N7 of 3A and in some cases also with the amino group of CMBL’s short linker. H-bonding is also observed between the two water molecules (distance = 2.3–2.9 Å).

### Higher order interactions between the RNA-molecules

RNA-CMBL complexes crystallized in five different space groups (Table 1). A comparison of the structures allowed us to observe different ways in which the RNA molecules interact. The common contacts observed in all of the structures involve the end-to-end stacking of RNA duplexes, resulting in the formation of pseudocontinuous helices. Other interactions engage the flipped out 4G and 5C residues. In all models, the two consecutive residues stack one above another and tend to base pair with the corresponding residues of an adjacent RNA molecule. One type of interaction includes the typical Watson–Crick pairing and formation of G–C and C–G pairs, which can either fill the voids between duplexes (as in seleno modified CAG(Se)-CMBL3a model) or form short insertions in the pseudo-infinite helical stack (CAG-CMBL4) (Supplementary Figure S2A, B). In more complex cases, in addition to the Watson-Crick pairs, triplex-like interactions are observed between the bulged-out nucleotides (CAG-CMBL3b and CAG-CMBL3a). The 4G and 5C residues can enter the major grove and form additional contacts with the canonical G–C and C–G pairs (Supplementary Figure S2B). Alternatively, in the CAG-CMBL3a(I), the flipped out 4G and 5C residues form an i-motif like structure consisting of C–C+ and G–G pairs (Supplementary Figure S2C). The two nucleobases engage in H-bonding via their Watson–Crick edges are oriented *in trans* relative to the *N*-glycosidic bonds, resulting in the parallel orientation of the RNA chains. In some cases, the flipped out 4G and 5C residues do not form any H-bond interactions with neighboring RNA molecules. Such a CG stack can overlay a triplex-like motif or it can have no further ordered interactions.

### Temperature melting and biochemical analysis

Initial measurements of the effect of CMBL compounds on the CAG repeats were performed with CMBL3a on two RNA oligomers: UCAACAGUUGA and GCAGCAGC, showing an increase in the melting temperature (Supplementary Figure S3). More detailed studies were performed on the GCAGCAGC oligomer that was chosen for the crystallization. All of the (GCAGCAGC)_2_–CMBL complexes showed an increase in thermal stability compared to the unliganded RNA (Table 2). In the presence of CMBL3a, the duplex is the most stable, having a melting temperature *T*_M_ nearly 20°C higher. For CMBL3b and CMBL4, the increases are approximately 6°C and 8°C, respectively. The UV-melting experiments clearly indicate that all the CMBL molecules bind and stabilize RNA containing CAG repeats.

**Table 2.** The thermodynamic parameters of RNA and RNA-CMBL complexes

	Average of curve fit				*T*_M_^−1^ versus log C_T_ plots			
	–Δ*H* (kcal/mol)	–Δ*S* (eu)	–Δ*G*_37_ (kcal/mol)	*T*_M_^a^(°C)	–Δ*H* (kcal/mol)	–Δ*S* (eu)	–Δ*G*_37_ (kcal/mol)	*T*_M_^a^ (°C)
(GCAGCAGC)_2_	39.1±4.6	109.5±15.4	5.2±0.1	33.1	38.6±2.0	107.7±6.7	5.2±0.1	33.0
^b^(GCAGCAGC)_2_-CMBL3a	68.0±2.4	191.1±7.3	8.7±0.6	51.5	42.2±2.4	110.8±7.6	7.8±0.1	53.6
^b^(GCAGCAGC)_2_-CMBL3b	55.0±10.0	157.8±31.0	6.1±0.5	39.3	34.6±4.6	92.1±15.0	6.0±0.3	40.1
^b^(GCAGCAGC)_2_-CMBL4	57.2±6.7	163.4±21.2	6.5±0.2	41.4	61.6±4.4	177.7±14.3	6.5±0.1	41.4

^a^Melting point was calculated at the oligomer concentration of 10^−4^ M.

^b^Thermodynamic parameters were calculated assuming saturating levels of ligand concentration.

The surface plasmon resonance (SPR) assay of CMBL3a binding was performed on (CNG)_9_ oligomers. The RNA immobilized on sensor surfaces clearly showed a significant and selective SPR response for (CAG)_9_ (Supplementary Figure S4A). No SPR responses were observed for (CGG)_9_ (Supplementary Figure S4B) and (CCG)_9_ (Supplementary Figure S4C). A decrease in the SPR response was observed for (CUG)_9_ (Supplementary Figure S4D). This indicates that CMBL3a interacts specifically with CAG repeats.

**Figure 3.**
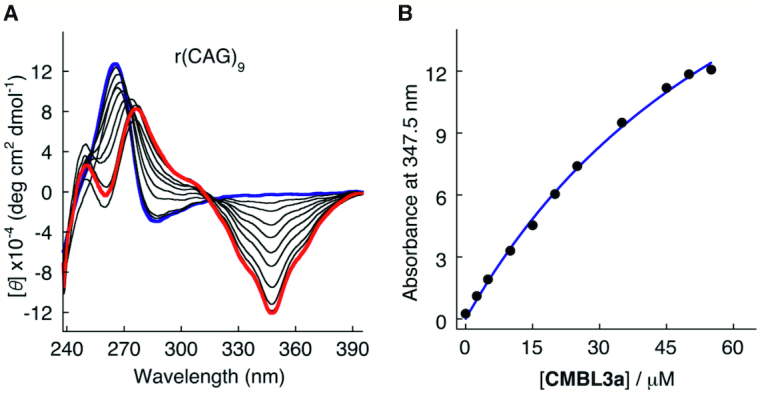
Biochemical analysis of CMBL3a interacting with CAG RNA repeats using circular dichroism. (**A**) CD spectra. (**B**) Ellipticity at 347.5 nm obtained from titration data were plotted against the CMBL3a concentration.

Circular dichroism (CD) spectra of (CAG)_9_ showed a clear conformational change upon CMBL3a binding. An induced negative CD band was observed at 347.5 nm. The molar ellipticity of the negative peak at 347.5 nm increased with the ligand concentration (Figure 3A, B). Based on the titration experiment, the apparent Kd was calculated to be 70.5 μM, assuming a 1:2 binding isotherm.

## DISCUSSION

The presented results are part of our search for compounds that would interact specifically with the pathogenesis-related RNA repeats. The described complexes of the cyclic bis-naphthyridines with CAG repeats are the first crystal structures of this type. The models show details of the ligand binding, local and nonlocal effects on the RNA structure and interactions with the solvent, which should be useful for developing therapeutics against TREDs. The structural results are supported by biochemical data.

### Effect of CMBL ligands on the structure of CAG repeats

A comparison of the models shows a consistent mode of binding of the CMBL ligands to the CAG RNA repeats. Structures of the complexes can be compared with the known crystal structures of native CAG repeats, which form fully double stranded A-RNA helices in which the A–A pairs interact *via* one weak N1···H-C2 H-bond, and the large purine rings are accommodated by shifting them towards the major groove of the helix (20). As a result, their Watson-Crick edges are exposed in the major groove and interact with a sulfate anion. The binding of CMBL causes large structural changes in the RNA. The adenines, instead of pairing with one another, engage in pairing interactions with naphthyridine moieties. As a consequence, one weak H-bond of the A–A pair is replaced by four H-bonds because each adenosine residue now forms a pair of bonds with the naphthyridine rings. The hydrogen bonding potential of adenosines is fulfilled, as in the canonical A–U pair, and the thermal stability of the pseudo canonical pair is increased compared to the native model. On the other hand, two C–G pairs become unpaired and flipped out of the helix. Upon a rough count, the overall number of H-bonds within the double helix remains the same upon binding the CMBL ligand.

The rearrangements around the adenosine residues are reflected by changes of the helix backbone conformation. The backbone torsion angles are changed, one adenosine of each pair has the ribose ring in the C2′-*endo* conformation and the flipped out C and G residues engage in intermolecular interactions. The CD spectra also show major changes when CMBL3a is added to the (CAG)_9_ oligomer, providing additional evidence of structural rearrangements, even though the spectroscopic measurements and crystallography were performed under different experimental conditions.

The flipping out of G and C residues is unexpected since the G–C pairs have been a constant feature in all the structures of the CNG repeats and are expected to be a major stabilizing factor in the duplex. Such extensive structural rearrangements would be difficult to predict by *in silico* approaches. Nevertheless, the UV-melting experiments indicate that the rearrangement caused by the ligand is thermodynamically favorable. The melting temperature of the RNA–ligand complex is consistently higher than for the sole RNA molecule, suggesting that stability is increased by the formation of adenosine-CMBL pairs. Removing the C–G pairs from the helix could relieve geometric constrains and allow optimal stacking interactions of the remaining nucleobases with the naphthyridine rings.

The flipping out of the C and G residues has consequences on the long-range ordering of the molecules because, as opposed to the native duplex, it has gained a considerable potential for cross-helix interactions, as can be observed in the crystal structures. In each of the five different crystal lattices, the externalized bases engage in intermolecular contacts, forming i-motifs or pseudohelices, in which the C and G residues form Watson-Crick base pairs and interact *via* ring stacking (Supplementary Figure S2C). Considering the proportion of C and G bases in CAG repeats, externalization of them creates a potential for cross-helix interactions that is comparable in energy to the intrahelix contacts.

### X-ray structures versus biochemical data

The biochemical data correlate very well with the crystallographic models. The SPR, thermal melting and CD spectroscopy indicate that CMBLs interact with the RNA molecules containing CAG repeats. The study using UCAACAGUUGA confirms that even a single CAG triplet is sufficient to bind the CMBL3a ligand. The X-ray structures show that all three ligands interact with the RNA in very similar manners, but the conformation of the bound CMBL3a is the least distorted from the idealized geometry. The asymmetric *trans* conformation of the long linker makes the ligand better adjusted to the binding site than the symmetric CMBL3b and CMBL4 (Supplementary Figure S5). This is consistent with the thermal melting observations that CMBL3a forms more stable complexes with the RNA than CMBL3b or CMBL4. The SPR data show the selectivity of CMBL3a towards RNA containing CAG repeats compared to other CNG triplets, even though the 1,8-naphthyridine heterocycle in CMBLs was designed to have the hydrogen bonding potential complementary to guanine (31). The clear signal is in agreement with the crystallographic results, which show the ligand binding the noncanonical A–A pairs not present in the other oligomers. CD spectra measured in solution also showed that CMBL compounds induce higher order changes in the RNA structure. The looped-out C and G residues observed in the crystal lattice give an indication of what could happen in the crowded environment of the aggregated RNA, characteristic of the trinucleotide repeat disorders.

The directness of CMBL’s interaction with the CAG repeats and the large-scale structural consequences raise expectations for possible therapeutic applications. Although more data is needed we suggest that the compounds would act in a way to bind the mRNA aggregated in the nuclear foci and outcompete the sequestered proteins, releasing them back into the intranuclear space. Taken together, the results constitute an early characterization of the first lead compounds in the search for therapy against TREDs.

### How to improve the properties of the CMBL ligands

Inspection of the crystal structures reveals a number of features that make the CMBL compounds bind well to their target. One is the cyclic arrangement of the two naphthyridines, which is well suited for the recognition of the noncanonical A-A pairs. It allows the CMBL ligand to bind simultaneously to the two adenosines located on the opposite RNA strands (Figure 1C). The binding orientation of the ligand molecule, with the short linker in the major groove and the long linker in the minor groove, is appropriate for specific interactions within the RNA duplex. The other orientation, with the longer linker in the major groove, seems improbable, since the naphthyridine rings would be shifted out of the core of the helix and would no longer be in a position to stack with the nucleobases.

When considering the factors determining the affinity and specificity of the CMBL ligands towards the RNA, one needs to take into account the following: (i) the aromatic rings of the ligands and their potential for stacking interactions, (ii) the ring edges and their potential to form H-bonds with the nucleobases and (iii) the linkers between the rings.

CMBL ligands make extensive stacking interactions within the RNA duplex; each naphthyridine moiety is inserted between two nucleobases. Much of the binding energy certainly comes from aromatic stacking, but such interactions are nonspecific. The base-pairing contacts are much more selective. The inner edge of the naphthyridine ring has the potential for three H-bonds: the two imine groups and one amine, evenly spaced, form an array of two acceptors and a donor (Figure 1A). Two of these, the donor and the neighboring acceptor, are engaged in pairing with the Watson-Crick edge of adenine. Four such H-bonds are formed for each ligand. Simultaneously base-pairing the two adenosines requires relative tilting of the naphthyridine rings. For this to be possible, the linker on the side of the minor groove needs to be sufficiently long. In all three types of the CMBL ligands the linkers have similar lengths, which seem to be just sufficient. The linker on the side of the major groove is short but adequate to span the height of the RNA step. This linker could possibly be extended by one or two covalent bonds. The crystal structures show that the shorter linker can be labile, showing two alternative conformations. The linker also has a H-bonding potential, as it binds one of the adenines *via* a water molecule (Figure 2A). A possible way to improve the ligand selectivity and affinity would be to stabilize the conformation of the linker and augment it by a function that would replace the water molecules and bind the adenines directly. Another possibility is to connect two CMBL ligands *via* the shorter linker to make a ligand that would target two A–A pairs simultaneously. The crystal structures show the liganded adenosines come into direct contact as the C and G residues are looped out of the helix. A ligand that would stabilize such a state should be more specific than two ligands acting separately. One of approaches toward this strategy has been reported recently (47). In the course of drug development, the linkers could be functionalized to improve the ligands’ pharmacokinetic properties or cellular uptake.

## DATA AVAILABILITY

Atomic coordinates and structure factors for the reported crystal structures have been deposited with the Protein Data bank under accession numbers 6QIQ, 6QIR, 6QIS, 6QIT, 6QIV.

## Supplementary Material

gkz832_Supplemental_File

## Notes

In memory of Prof. Włodzimierz Krzyżosiak, who inspired us in our research on trinucleotide repeats-related disorders.
