# Supplementary material for: Structural insights into synthetic ligands targeting A–A pairs in disease-related CAG RNA repeats
Source: Nucleic Acids Res. 2019 Sep 30;47(20):10906–13. doi: 10.1093/nar/gkz832 (PMC6847237; doi:10.1093/nar/gkz832)
Supplement: gkz832_Supplemental_File [file gkz832_supplemental_file.pdf]

SUPPLEMENTARY DATA

Structural insights into synthetic ligands targeting A-A pairs in disease-related CAG RNA repeats

Sanjukta Mukherjee[1†], Leszek Błaszczyk[2†], Wojciech Rypniewski[2], Christoph Falschlunger[3], Ronald Micura[3], Asako Murata[1], Chikara Dohno[1], Kazuhiko Nakatani[1*], Agnieszka Kiliszek[2*]

[1]Department of Regulatory Bioorganic Chemistry, The Institute of Scientific and Industrial Research, Osaka University 8-1 Mihogaoka, Ibaraki 567-0047, Japan

[2]Institute of Bioorganic Chemistry, Polish Academy of Sciences, Noskowskiego 12/14, 61-704 Poznan, Poland

[3]Institute of Organic Chemistry and Center for Molecular Biosciences Innsbruck CMBI, Leopold-Franzens University, Innrain 80-82, Innsbruck 6020, Austria

[†] These authors contributed equally to this work

* Correspondence should be addressed to A.K. (kiliszek@ibch.poznan.pl) or K.N. (nakatani@sanken.osaka-u.ac.jp).

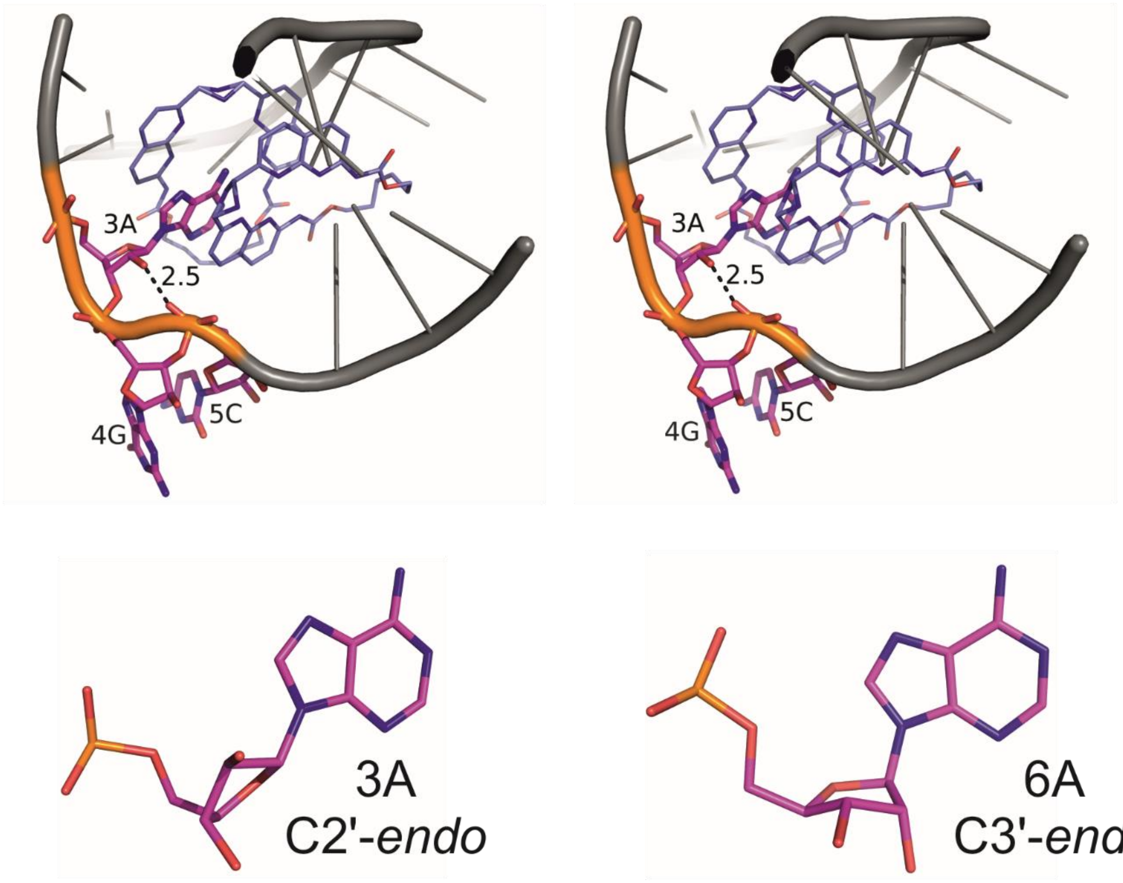

Figure S1. The rearrangement of the RNA chain (orange) as a result of flipping out of the 4G and 5C residues and C2'-*endo* conformation of the 3A ribose (stereo view). The hydrogen bond between 2'OH group of 3A and oxygen atom of 5C is marked as dashed line.

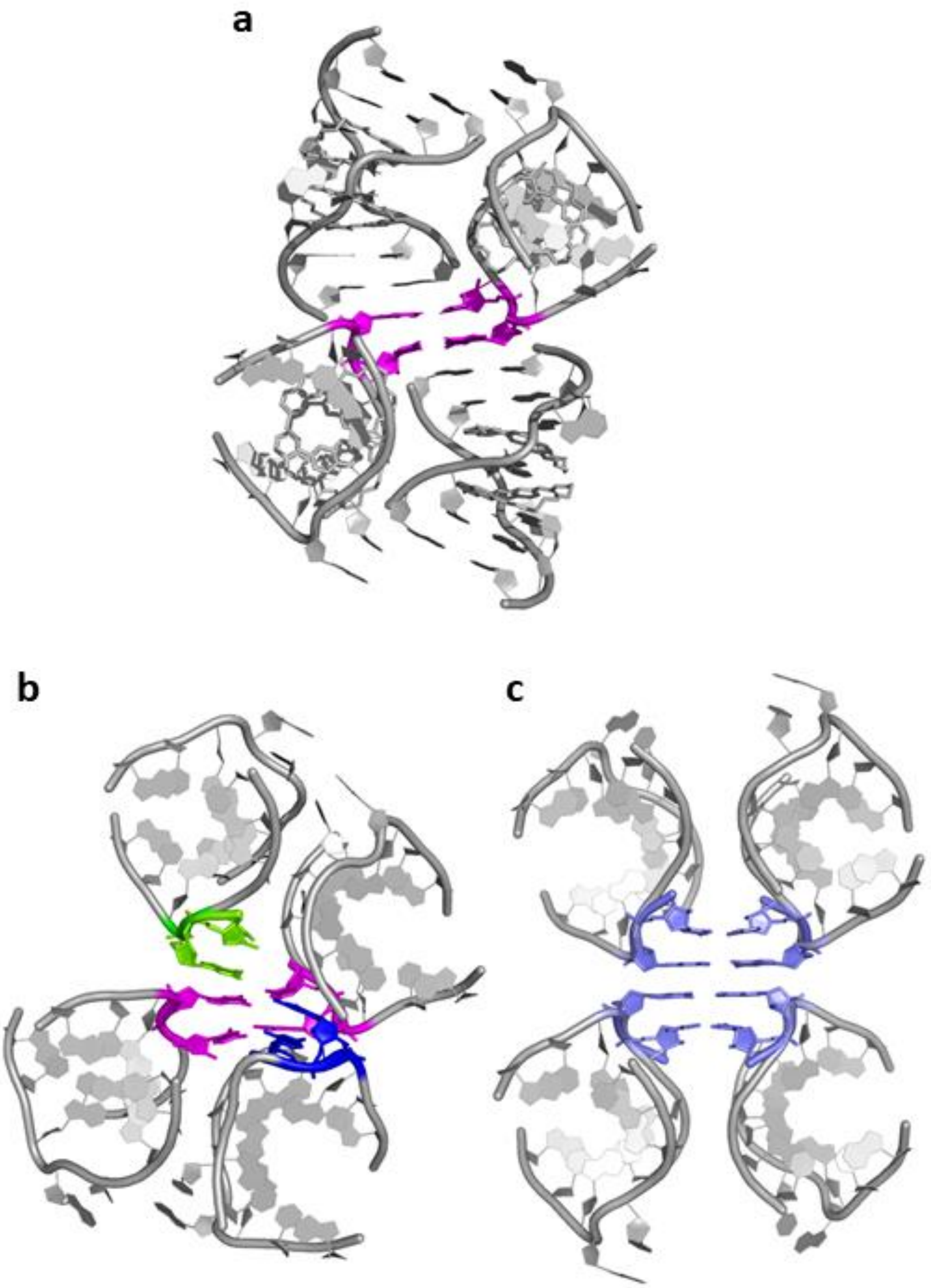

Figure S2. Higher order interactions in the crystal lattice of RNA-CMBL complexes. **a**, Extruded 4G and 5C residues (*purple*) are integrated into the pseudo-infinite helix in the crystal lattice of the CAG-CMBL4 structure. **b**, A complex interaction involving four RNA duplexes, including Watson-Crick pairing (*purple*) triplex (*navy*) and stacking (*green*), in the CAG(Se)-CMBL3a model. **c**, An i-motif like (*blue*) in the CAG-CMBL3a(I) structure.

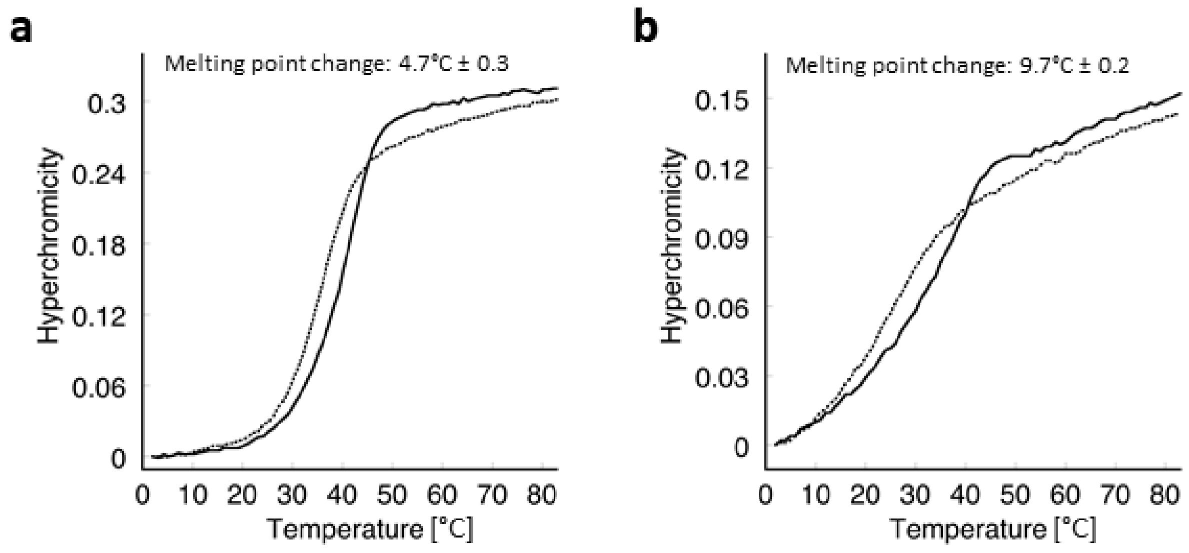

Figure S3. Thermal denaturation profiles of the CAG-containing RNA. **a**, $(UCAACAGUUGA)_2$ and **b**, $(GCAGCAGC)_2$. Dashed and bold lines represent the melting curve without and with CMBL3a, respectively.

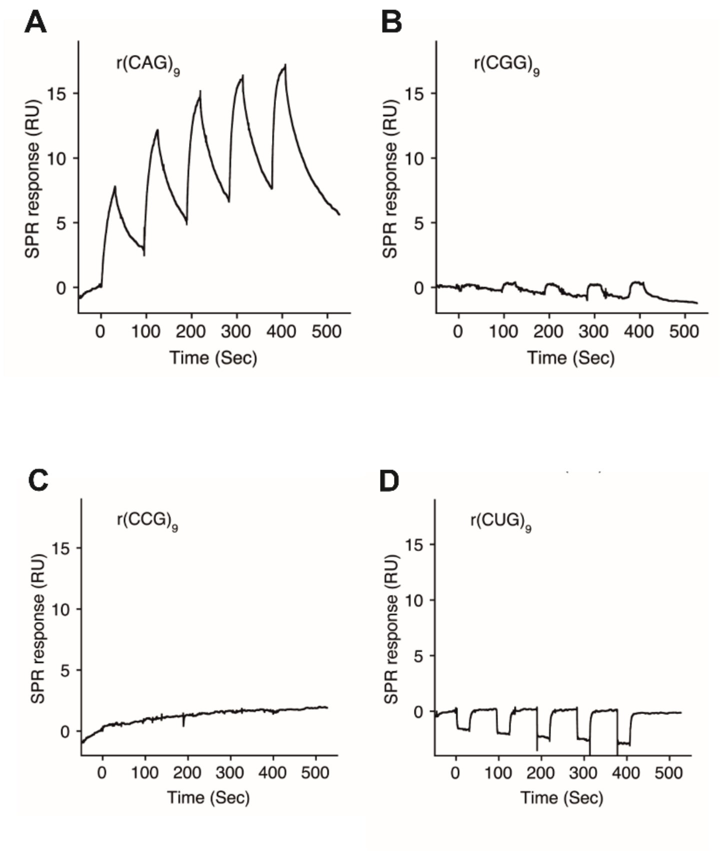

Figure S4. Biochemical analysis of CMBL3a interacting with CNG RNA repeats using Surface Plasmon Resonance. **(A-D)** SPR response as CMBL3a is added at increasing concentrations to immobilized RNA.

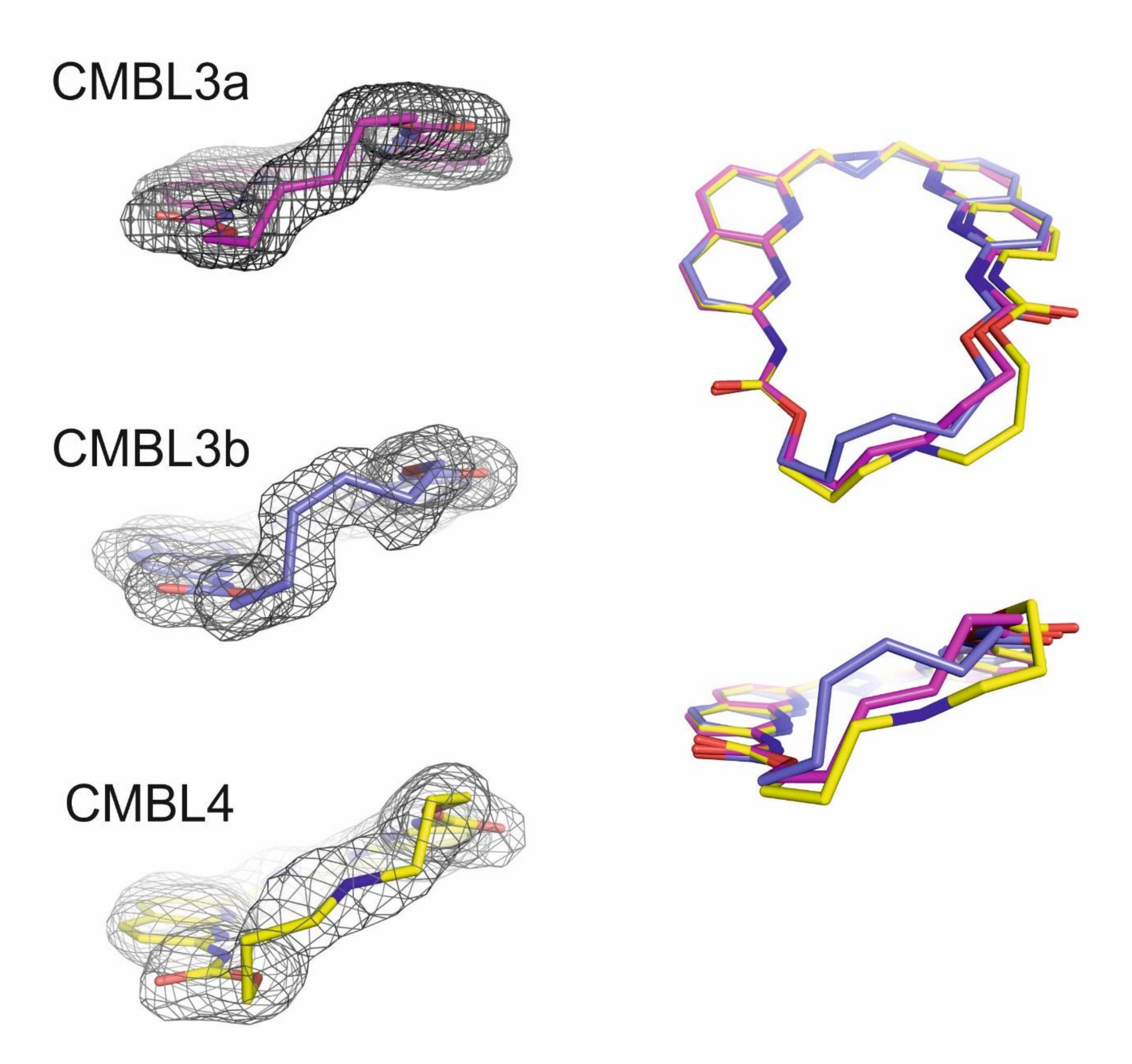

Figure S5. Conformation of the longer linker in the CMBL ligands. **Left panel**, The $2F_o$-F_c electron density map of the CMBL ligands countered at 1σ (grey). **Right panel**, Superposition of the structures of CMBL ligands showing differences in the conformation of the longer linker.
